# Bone-on-a-Chip Systems for Hematological Cancers

**DOI:** 10.3390/bios15030176

**Published:** 2025-03-09

**Authors:** Gül Kozalak, Ali Koşar

**Affiliations:** 1 Faculty of Engineering and Natural Sciences, Sabancı University, Istanbul 34956, Turkey; gul.kozalak@sabanciuniv.edu; 2 Center of Excellence for Functional Surfaces and Interfaces for Nano Diagnostics (EFSUN), Sabancı University, Istanbul 34956, Turkey; 3 Turkish Academy of Sciences (TÜBA), Çankaya, Ankara 06700, Turkey

**Keywords:** bone-on-a-chip, hematological cancers, disease modelling, drug imaging

## Abstract

Hematological malignancies originating from blood, bone marrow, and lymph nodes include leukemia, lymphoma, and myeloma, which necessitate the use of a distinct chemotherapeutic approach. Drug resistance frequently complicates their treatment, highlighting the need for predictive tools to guide therapeutic decisions. Conventional 2D/3D cell cultures do not fully encompass in vivo criteria, and translating disease models from mice to humans proves challenging. Organ-on-a-chip technology presents an avenue to surmount genetic disparities between species, offering precise design, concurrent manipulation of various cell types, and extrapolation of data to human physiology. The development of bone-on-a-chip (BoC) systems is crucial for accurately representing the in vivo bone microenvironment, predicting drug responses for hematological cancers, mitigating drug resistance, and facilitating personalized therapeutic interventions. BoC systems for modeling hematological cancers and drug research can encompass intricate designs and integrated platforms for analyzing drug response data to simulate disease scenarios. This review provides a comprehensive examination of BoC systems applicable to modeling hematological cancers and visualizing drug responses within the intricate context of bone. It thoroughly discusses the materials pertinent to BoC systems, suitable in vitro techniques, the predictive capabilities of BoC systems in clinical settings, and their potential for commercialization.

## 1. Introduction

Hematological cancers constitute a group of malignancies originating from cells in the blood, bone marrow, and lymph nodes. These cancers are broadly categorized into three principal groups: leukemia, lymphoma, and myeloma. Leukemia arises specifically from blood and bone marrow cells and can be further classified into lymphocytic and myelogenous types. Lymphoma, on the other hand, originates in the lymph nodes and is divided into Hodgkin and non-Hodgkin subtypes. Multiple myeloma is distinguished by the proliferation of a specific variant of plasma B cells within the bone marrow, leading to the production and secretion of monoclonal immunoglobulins [1]. In the treatment of hematological cancers, the employed specific drug regimen can differ significantly from one condition to another. However, it is important to note that a variety of chemotherapeutic agents are commonly utilized, including cytostatic drugs, alkylating agents, alkaloids, antibiotics, tyrosine kinase inhibitors, and antimetabolites [2]. These agents play a crucial role in inducing the death of cancer cells, contributing to effective patient management. The majority of patients ultimately develop resistance to pharmacological treatments, necessitating the exploration of alternative medications or drug combinations to address this challenge [1]. Regrettably, this results in a cyclical dilemma that complicates the selection of the most suitable treatment options. Traditional two-dimensional (2D) in vitro cultures are insufficient for long-term studies and do not provide a reliable foundation for clinical extrapolation, as they do not encompass all the growth factors present in the in vivo environment [3]. Moreover, the absence of certain in vivo criteria, such as spatial arrangement, shear stress, and geometric simulation in both 2D and 3D cell cultures, complicates the process of translating findings from murine models to human applications. The findings reveal that the average cost to transition a potential drug from the laboratory to clinical application is approximately USD 2.8 billion [4,5]. As such, there is a pressing need for practical bedside tools that can effectively predict patient responses to medications and inform the development of tailored treatment strategies. To effectively determine the most appropriate treatment options for patients with hematological cancers and to successfully apply personalized therapy approaches in clinical practice, it is crucial to focus on the following objectives: (1) to streamline the time required to utilize tools that identify effective treatment strategies, (2) to proactively assess both drug resistance and sensitivity, and (3) to identify cellular biomarkers that may indicate potential side effects associated with the treatment [6].

Organ-on-a-chip (OoC) systems represent a significant advancement in microfluidic technology, integrating principles from both biology and engineering to accurately simulate human tissues and organs at the microscale [7]. These innovative platforms leverage the microsystem technology to design OoCs that exhibit specific biochemical and physiological properties tailored to emerging research needs [8,9]. This technology facilitates the development of intricate microfluidic channels, which are complemented by miniature actuators embedded within the OoC systems [10]. The chips utilized in OoC systems have complex microchannel networks that adeptly manipulate minute volumes of solutions. The development of human disease models using OoC systems not only enhances the understanding of various diseases but also plays a crucial role in the formulation of effective therapeutics. The choice of an appropriate OoC system is guided by the biochemical and physiological attributes of the biological system intended for simulation. While there have been advances in OoC systems, the research on bone-on-a-chip (BoC) models in the context of hematological cancers remains limited. BoC systems provide a cutting-edge platform that replicates the microenvironment of bone tissue in vitro, utilizing advanced microfluidic and organotypic technologies. These systems facilitate the co-culture of bone cells, including osteoblasts, osteoclasts, osteocytes, and bone marrow stromal cells, within microfluidic chips. This setup enables real-time observation of interactions among cells and between cells and the extracellular matrix [11]. Additionally, BoC systems are capable of simulating the mechanical stresses and biochemical gradients inherent to natural bone tissue. These capabilities enhance the accuracy of disease modeling, particularly for hematological cancers, osteoporosis, and bone metastasis. The advances in BoCs over the years are summarized in chronological order in Table 1. Existing studies [12,13,14] have mainly focused on the characterization of bone tissue and cannot adequately model hematological cancers.

The intricate nature of bone tissue, characterized by various cell types and matrix components, along with the diverse symptoms present in hematological cancers, poses significant challenges in predicting the treatment efficacy. While 2D cultures offer straightforward cell sheets and 3D cultures provide more intricate structures, BoC systems excel in simulating a dynamic fluidic microenvironment, along with realistic cell–cell and cell–matrix interactions. These platforms are capable of accurately recreating the complex signaling interactions present in the bone marrow microenvironment, thereby enhancing the predictability of drug responses, particularly in hematological cancers. Moreover, BoC systems can replicate natural mechanical forces, such as fluid shear stress and mechanotransduction, as well as biochemical gradients akin to those found in bone tissue. These characteristics provide significant benefits for both cell differentiation and disease modeling. Traditional animal models may not fully capture human physiology, whereas BoC platforms utilize human cells and tissues, yielding more precise and reliable results in terms of drug efficacy and toxicity. Additionally, these systems facilitate the development of personalized disease models with the use of patient-derived cells, thereby contributing to the advancement of personalized medicine. To enhance the clinical predictive capabilities of BoC platforms, the integration of biosensors can facilitate dynamic and real-time data collection. This approach can significantly improve the accuracy and reliability of patient assessments. It is, therefore, crucial to develop BoC systems that are designed for bedside application and proficient in characterizing the bone microenvironment in vivo. Such systems will enable us to anticipate drug responses in the context of hematological cancers, mitigate the risk of drug resistance, and facilitate the implementation of personalized therapeutic approaches.

In this review, we present an overview of the various types of hematological cancers and the currently employed treatment regimens. We also detail the critical factors necessary for accurately describing bone physiology within BoC systems and identify the most appropriate techniques for the construction of BoC models. Moreover, we summarize the hematological cancer models that have been developed to date, emphasizing the techniques and biomaterials essential for effective modeling. Furthermore, we explore the research surrounding drug development and imaging strategies pertinent to hematological cancers, with a particular focus on the role of biosensors. Finally, we provide an overview of the microfluidic platforms that have been commercialized to date and discuss the potential of BoCs for advancements in clinical and commercial applications. An outline table of the review is provided in Table 2.

## 2. The Biological Landscape of Hematologic Cancers

Blood serves as the vital fluid that delivers oxygen and essential nutrients to every cell in the human body. Blood cells are generated through the processes of division, proliferation, and differentiation of pluripotent stem cells located in the bone marrow. Lymphoid stem cells are responsible for the formation of T lymphocytes, B lymphocytes, and natural killer (NK) cells, while myeloid stem cells give rise to erythrocytes, platelets, monocytes, and granulocytes. Hematologic cancers can emerge at any stage of lymphocyte development, often as a result of malignant transformation or mutations occurring within the stem cells [19]. Hematologic cancers arise from the uncontrolled proliferation of abnormal cancer cells rather than the body’s normal immune response to infections. For diagnostic purposes, a comprehensive range of standard tests is employed. These tests typically include biochemical analyses, hemograms, urine examinations, immunophenotyping, bone marrow aspirates, cytogenetic and molecular analyses, along with various imaging techniques. Leukemia is primarily classified into two principal categories based on the origin of the cells involved: lymphoid and myeloid. Each of these categories is further divided into acute and chronic types according to the progression rate of the disease [20]. Chronic leukemias are characterized by a slow progression from mature cells, while acute leukemias exhibit rapid development from immature cells [21]. Additionally, leukemias can be subdivided based on specific chromosomal abnormalities and morphological characteristics that result from lineage selection [22]. Acute lymphoblastic leukemia (ALL) is recognized as the most prevalent type of leukemia [23]. B-cell ALL is predominantly found in pediatric populations, whereas T-cell ALL is more frequently observed in adults [24]. ALL is characterized by loss of appetite, weight loss, and night sweats. The treatment regimens for ALL typically include daunorubicin, vincristine, prednisone, and asparaginase (Table 3). Chronic lymphocytic leukemia (CLL) is generally diagnosed in older patients and is primarily associated with the formation of B-cell tumors [25]. Fatigue, night sweats, and susceptibility to infections are common in CLL. Therapeutic options for CLL include ibrutinib, zanubrutinib, acalabrutinib, obinutuzumab, and rituximab (Table 3). Acute myeloid leukemia (AML) is a well-recognized type of myeloid leukemia in adults; bleeding under the skin and skin rashes can be seen. AML treatment approaches include cytarabine, daunorubicin, and idarubicin (Table 3) [26]. Chronic myeloid leukemia (CML) is characterized by the presence of the Philadelphia chromosome and generally exhibits a gradual progression [27]. CML is characterized by a sensation of fullness in the abdominal area, as well as discomfort in the left upper quadrant, which can be attributed to splenic enlargement. The treatment options available for CML include imatinib, dasatinib, and nilotinib (Table 3) [28].

Lymphomas constitute a category of malignancies that originate from malignant lymphocytes, primarily located within the lymph nodes [21]. These abnormal lymphocytes can potentially spread to various regions of the body through the lymphatic system [19]. Lymphomas are classified into two primary groups: Hodgkin lymphoma (HL) and non-Hodgkin lymphoma (NHL) [2]. HL is further subdivided into classical and non-classical forms, whereas NHL is categorized based on the types of involved lymphocytes, including B cells, T cells, and natural killer (NK) cells [2]. HL is derived from B lymphocytes and is associated with a notably high cure rate [29]. A hallmark feature of HL is the presence of multinucleated Reed–Sternberg cells [30]. HL presents symptoms of unilateral, painless lymph node enlargement in the neck area, fever, night sweats, and weight loss. The chemotherapeutic agents commonly employed in the treatment of HL encompass doxorubicin, bleomycin, vinblastine, and dacarbazine (Table 3) [31]. In contrast, Reed–Sternberg cells are not present in NHL, which is related to genetic mutations that affect the development of T, B lymphocytes, and NK cells. It is characterized by a feeling of pressure and organ involvement accompanied by lymphadenopathy (neck, axillary, and inguinal). Within NHL, B-cell lymphoma constitutes the most prevalent form, with diffuse large B-cell lymphoma (DLBCL) recognized as the most aggressive subtype [32]. Lymphomas that remain localized within the lymph nodes are known as nodal lymphomas, while those that spread to other organs through the circulatory system are called extranodal lymphomas [33]. Therapeutic strategies for NHL generally involve using cyclophosphamide, doxorubicin, vincristine, and prednisone (Table 3). In cases where CD-20^+^ B cells are identified, rituximab is often incorporated into the treatment regimen (Table 3) [34].

Multiple myeloma (MM) is a complex hematological malignancy characterized by the abnormal proliferation of malignant plasma cells within the bone marrow [35]. These malignant cells originate from mutations that occur during the differentiation of B cells into plasma cells, leading to the production of abnormal antibodies known as M proteins [36]. The detection of these M proteins in urine serves as a key diagnostic marker for the disease [1]. The progression of MM typically encompasses several stages: monoclonal gammopathy of unknown significance (MGUS), smoldering multiple myeloma (SMM), and active multiple myeloma [37]. Patients may experience a range of complications due to the dysregulated secretion of M proteins, which can manifest as hypercalcemia, renal impairment, anemia, and bone lesions, collectively referred to as the “CRAB” symptoms [38]. For the treatment of MM, first-line therapy commonly involves a combination of bortezomib, lenalidomide, and dexamethasone, which is effective in managing the disease (Table 3) [39].

## 3. Simulating Bone in Microfluidic Chip Platforms

**Figure 1:**
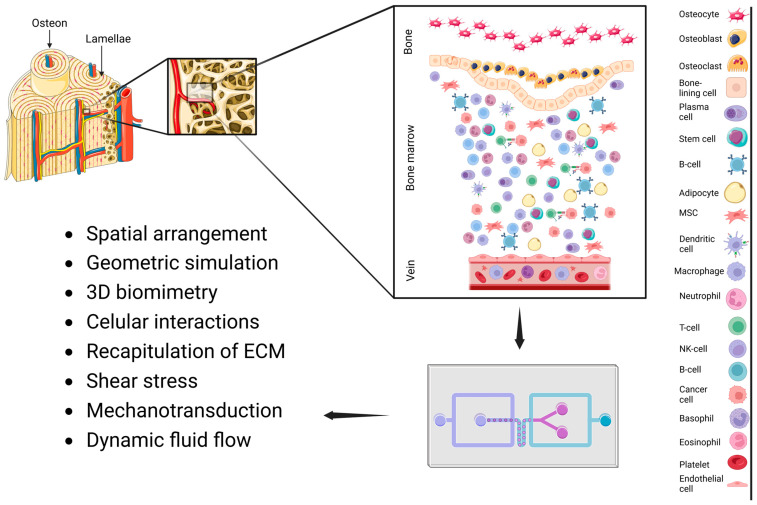
Schematization of bone delineation in microfluidic chip platforms.

The skeletal system is primarily composed of bones, which play a crucial role in maintaining the body’s structural integrity while also encompassing elements of the nervous and circulatory systems, thereby facilitating muscle movements. The bone tissue is continually exposed to various mechanical influences, including tensile and compressive stresses, shear stresses induced by fluid movement, and stiffness from the extracellular matrix (ECM) [40]. Long bones consist of spongy (trabecular) bone and cortical bone [41]. The bone marrow is recognized as the principal site for the development of both blood and immune cells [42]. The osteons are sophisticated structures composed of lamellae that incorporate circulatory and nervous system elements known as Haversian canals (Figure 1) [17]. The processes of proliferation, differentiation, and apoptosis in bone cells (osteoblasts, osteocytes, and osteoclasts) are regulated by intercellular interactions and their relationship with the ECM [43]. Osteoclasts are multinucleated cells that differentiate from hematopoietic stem cells and are activated by osteocytes and osteoblasts to play a crucial role in bone resorption and remodeling (Figure 1) [44]. Osteoblasts, which originate from mesenchymal cells (MSC), can differentiate into osteocytes when they become embedded in lacunar spaces (Figure 1) [45]. During this process, osteoblasts perform a vital function in enhancing the hardness of the osteoid tissue by secreting various components of the bone ECM along with essential minerals [46]. The overall process can be delineated into three primary stages: (1) the proliferation of osteoblasts alongside the secretion of collagen and calcium-phosphate crystals; (2) a reduction in osteoblast proliferation, accompanied by an increase in osteoclast activity and the secretion of alkaline phosphatase (ALP) as the bone matrix matures; and (3) mineralization, which involves a decrease in ALP synthesis and the subsequent differentiation of osteocytes [45,47]. Microfluidic devices are promising platforms for enabling precise control over parameters such as media perfusion, interstitial flow, and shear stress through the use of microchannels (Figure 1) [45]. This technology effectively simulates a physiological three-dimensional environment, facilitating the study of continuous mechanical loads and dynamic fluid flows, which are vital stimuli for bone development (Figure 1) [48]. Moreover, microfluidic chip platforms are highly versatile and allow for the collection of comprehensive data while minimizing the use of samples and materials [17]. They offer the capability to culture cells in distinct compartments, thereby supporting spatial and temporal separation of various cell types [49]. To effectively recapitulate BoC systems at the microscale, it is imperative to select appropriate materials and adapt designs that are tailored to the specific tissue in question.

BoC platforms should be designed to accurately represent the geometric structure and spatial arrangement of cells specific to the bone tissue. Recent studies explored the innovative use of bone scaffolds or actual bone as molds to create biologically relevant simulations [13,50]. The resulting 3D biomimetic bone platforms demonstrated a strong capacity to replicate the trabecular structure of bone in vivo, as confirmed by nanocomputed tomography imaging [13]. Moreover, a network of osteocytes was developed within a microfluidic system featuring porous microbeads to model the lacunocanalicular structure associated with bone remodeling [51]. The trabecular bone model was illustrated on a device containing demineralized bone paper, demonstrating that bone remodeling occurred due to cell contact and paracrine interactions [52]. A recent study on osteonecrosis revealed that osteocytes treated with bisphosphonates had an adverse impact on osteoblast proliferation and bone formation when exposed to mechanical and inflammatory signals [53]. Another recent study examined bone remodeling in a scaffold-free three-dimensional environment and reported that MSCs successfully differentiated into osteoblasts [54]. The introduction of monocytes into the co-culture facilitated the fusion of these cells with osteoclasts [54]. The microfluidic system employed in the related study demonstrated its suitability for drug development, allowing for cell culture for 35 days [54]. Additionally, calcium was identified in osteocyte and osteoblast cells that were printed to investigate their functions and the effects of fluid shear stress on bone remodeling [55].

The bone tissue maintains a delicate balance between cell formation and destruction, influenced by mechanical forces such as tension and compression during movement. This process of mechanotransduction aligns bone cells according to the direction of the applied force vector, subsequently enhancing osteogenesis [56,57,58]. For BoCs to accurately model bone behavior, the incorporation of these naturally occurring tension and compression movements is essential, requiring careful consideration of the intensity and timing. The stiffness of the bone tissue is typically in the range of 13–18 GPa, providing the necessary mechanical support for the proliferation and differentiation of bone-forming cells [59,60,61]. For instance, a bone scaffold that was decellularized using a microfluidic system and supplemented with collagen and calcium achieved a notable hardness of 2 GPa [13]. In BoC systems, the degree of mineralization is also an important indicator of tissue stiffness, which is measured by Young’s modulus (Pa) [62]. A study reported that Young’s modulus of bone matrix was 3.5 MPa before mineralization and increased to 4.6 MPa following the process [63]. However, it is important to note that a significant limitation in BoC systems is that the chip must be opened to achieve measurements of Young’s modulus of the bone tissue [62]. Furthermore, it was demonstrated that the shear stress positively influenced osteogenic differentiation [55]. In a BoC system designed with robotic printing, where osteoblast and osteocyte microarrays were in communication, calcium response as a result of mechanical stimulation was detected [55]. Specifically, preosteoblastic cells cultured in a microfluidic system at a flow rate of 50 μL/min for seven days exhibited a greater proliferation than those in a static culture [64]. Notably, intermittent fluid shear stress applied within the microfluidic system was shown to enhance the osteogenic differentiation of MSCs [12]. However, it is important to recognize that an excessive increase in the fluid flow rate may hinder cell attachment.

The integration of biosensors into microfluidic chips facilitates real-time monitoring of biomarkers present in the cultivated tissue without the need to open the chip [65]. Biosensors on this task can be classified into three categories: optical, mechanical, and electrical, based on their response to stimuli [66]. Optical sensors identify light stimuli produced during biological reactions and are particularly effective for measuring parameters such as pH, glucose, lactate concentrations, and gases, including oxygen or carbon dioxide, within the chip environment [67]. Mechanical sensors evaluate the mechanical properties of cells, including the stress, strain, mass of biomolecules, and force and displacement on the chip [68]. Electrical sensors are designed to detect biological reactions through electrical means. Among these, transepithelial electrical resistance (TEER) sensors have been widely employed in various platforms to assess diverse biomolecules based on impedance, pH, and electrochemical affinity [69]. There are several biological methods for detecting tissue biomarkers following growth on BoC platforms, summarized in Table 4.

Bone formation and resorption are primarily governed by the activities of osteoblasts and osteoclasts, while osteocytes play a crucial role in maintaining the balance between these processes [17]. To effectively monitor the interactions among these three cell types in BoC systems, various biomarkers, including Osteocalcin, Col1, and BSP, were identified through immunohistochemical methods [70,71]. In a recent study focusing on the communication mechanisms between osteoblasts and osteocytes through calcium fluctuations, Fluo-4 fluorescent dye was employed [55]. Furthermore, Alizarin red and von Kossa dyes have been widely utilized in BoC systems, highlighting their significance in this field [12,72]. Cells can be visualized through the application of various fluorescent dyes in conjunction with a fluorescent microscope. The nucleus of the cell serves as a critical source of information regarding cell morphology and polarization. Staining agents such as DAPI and Hoechst 33342 are commonly employed for this purpose [76]. The states of the nuclei can be quantitatively assessed using the nuclear shape index formula, expressed as 4π × area/perimeter [73]. This methodology was integrated into the analytical processes of recent BoC studies that investigated osteogenesis [18]. The ELISA method serves as a valuable tool for evaluating cell secretions within BoC systems. For instance, Ma et al. employed this method to monitor the cytokines TNF-α and IL-6, assessing the impact of drug application on cell proliferation in a bone marrow chip model [74]. Another study focusing on bone remodeling utilized ELISA to quantify the concentrations of osteoprotegerin (OPG) and receptor activator of nuclear factor-kappa B ligand (RANKL), providing an understanding of the interactions between osteoblasts and osteoclasts [52]. Various biomolecules can be detected through enzymatic reactions using BoC models. ALP is an important indicator for assessing the development of osteoblasts, while tartrate-resistant acid phosphatase (TRAP) is utilized to identify the presence of osteoclasts [62]. For example, ALP was effectively employed to evaluate osteogenic differentiation with a BoC platform that explored osteoporosis [18]. Lipids in BoC systems are identified using dyes such as Nile Red and BODIPY derivatives. Nile Red is often favored in BoC models due to its exceptional fluorescence properties [62]. Additionally, BODIPY derivatives, which exhibit fluorophore characteristics, were found to be applicable in BoC systems [75]. In BoC systems, gene expression is typically assessed through mRNA analysis, which necessitates the opening of the chip. For the evaluation of osteogenesis, mRNA levels of RUNX2, ALP, Col1, BSP2, and osteocalcin were examined [18,70]. In the context of osteoclastogenesis, the mRNA levels of TRAP, CTSK, MMP-9, RANK, OPG, and RANKL serve as important indicators [18]. Furthermore, for the assessment of vascularization, the analysis of the ratios of VEGFR1 and VEGFR2 mRNA was conducted [50]. These detection techniques can be developed thanks to the biosensor technology and seamlessly integrated into BoC platforms. This integration has the potential to accelerate the transition from fundamental research to clinical applications by providing accurate and precise measurements while capitalizing on the miniaturization of the devices.

## 4. Materials and Manufacturing Processes for Fabrication of BoC Platforms

The materials selected for fabricating BoC platforms must fulfill several critical criteria. They should effectively support cell culture, maintain non-toxicity, demonstrate adequate permeability, and provide transparency for microscopic examination. Some materials that were used with biomaterials to represent the bone microenvironment are shown in Table 5. Elastomeric polymers are recognized for their significant advantages in BoC platforms, primarily due to their ease of processing and cost-effectiveness [77]. Among these materials, polydimethylsiloxane (PDMS) is the most widely used elastomer, noted for its exceptional elasticity and stability, which stem from the Si–O bonds in its structure [78]. In addition to these properties, PDMS plays a crucial role in microfluidic systems owing to its favorable optical characteristics, gas permeability, and biocompatibility [79]. PDMS-based microfluidic chips can be effectively fabricated through various accessible and standard methods, including soft lithography, laser engraving, and injection molding. However, it is important to consider some limitations associated with PDMS. Its incompatibility with certain organic solvents might result in undesirable outcomes within small-channel applications [80]. Furthermore, the potential for leakage of uncross-linked oligomers from PDMS could adversely affect cellular systems [81]. Another notable drawback is the adsorption of small molecules by PDMS, which poses challenges in microfluidic drug research efforts [82]. It is important to note that the propensity of PDMS to adsorb hydrophobic molecules, along with its sensitivity to solvents, presents limitations for long-term and commercial applications. Notably, related studies indicated that PDMS can interact with specific compounds, potentially impacting drug absorption and thereby diminishing the clinical predictability of preclinical safety assessments [83]. Moreover, the findings of the study by Moore et al., who compared the absorption capacities of MM cells with the drugs bortezomib and carfilzomib on PDMS and thermoplastic devices, indicated that PDMS exhibited a greater ability to absorb these pharmaceutical compounds [84]. PDMS is an effective material for laboratory-scale prototyping; however, it presents challenges related to cost and repeatability when considering large-scale production. Overall, while PDMS offers many advantages, careful attention should be given to these limitations for its effective application.

Thermoplastic materials are distinguished by their capacity to retain shape over extended periods. These materials are biocompatible, transparent, resistant to leaks, and exhibit minimal absorption of hydrophobic molecules [89]. The most commonly utilized thermoplastic materials for BoC are Polymethyl methacrylate (PMMA) and Cyclic Olefin Polymers (COC). PMMA is particularly regarded as an optimal choice for chip development due to its excellent biocompatibility, rigidity, durability, and thermal stability [90]. A key advantage of PMMA chips is their low permeability to small and hydrophobic molecules, which significantly enhances their potential applications in drug studies [91]. Furthermore, although PMMA chips demonstrate resistance to water, it is advisable to utilize adhesive bonding or thermal bonding methods to ensure effective sealing [90,92,93]. PMMA offers significant commercial viability and cost advantages due to its compatibility with industrial manufacturing techniques, such as injection molding. These attributes position PMMA as a highly suitable option for large-scale production and various commercial applications. PMMA is more advantageous for industrial manufacturing processes, as it offers both cost-effectiveness and scalability for commercial applications. COC chips are advantageous for large-scale production of BoCs due to their cost-effectiveness and durability. This thermoplastic material is often favored for applications related to devices, particularly those involving biosensors [94]. However, it is important to note that non-polar organic substances might compromise the structural integrity of COC. Furthermore, COC materials introduce challenges in drug research, which stem from their hydrophobic surfaces [95]. These challenges can be effectively addressed through surface modifications, such as photografting or UV-initiated polyacrylamide grafting techniques [96,97].

Glass is a noteworthy material for BoCs due to its unique properties. As an amorphous material, glass offers electrical resistance and optical transparency, making it suitable for various applications [98]. The processing of glass is typically informed using photolithography and wet or dry etching techniques [99]. One of the advantages of glass is its excellent resistance to chemicals and low background fluorescence [100]. Furthermore, glass chips facilitate cell adhesion and do not absorb small hydrophobic molecules, enhancing their functionality [101]. However, the use of glass in cell culture studies is somewhat restricted, as it lacks gas permeability [102]. Additionally, glass chips are often less favored because of their rigid structure and higher production costs [103].

Natural polymers derived from animals, plants, and microorganisms have increasingly been recognized as valuable alternatives for BoC systems [104]. Their notable properties, including flexibility, biocompatibility, and gas permeability, distinguish them in the field [105]. Examples of such natural polymers encompass collagen, elastin, keratin, various polysaccharides, and glycoproteins [106]. Synthetic derivatives provide effective solutions to the purification and sterilization challenges often associated with natural polymers. Moreover, the properties of synthetic biomaterials, such as strength and degradation rates, can be precisely modified through adjustments to molecular weight, concentration, and cross-linking mechanisms [107]. The utilization of natural polymers in BoC systems has a promising potential of advancing our understanding of hematological cancers and enhancing the impact of drug research.

Regarding the development of biocompatible materials for BoC systems, the implementation of the 3D bioprinting technology can effectively reduce repetition errors, allowing for the efficient mass production of standardized chips [108]. A variety of 3D bioprinting techniques—including stereolithography, inkjet printing, laser-assisted bioprinting, and fused deposition modeling—are applicable in the fabrication of microfluidic chips [109]. These methods facilitate the precise layer-by-layer patterning of cells and ECM components that constitute the complex tumor microenvironment (TME) on microfluidic chips [110]. 3D-bioprinted scaffolds can be further enhanced by incorporating Bone Morphogenetic Protein (BMP), which promotes bone formation; Vascular Endothelial Growth Factor (VEGF), which supports vascularization; and platelet-rich fibrin, which facilitates the differentiation of MSCs [111,112]. Moreover, additive manufacturing and 3D bioprinting technologies act as key factors for developing microfluidic chips that can be utilized in disease modeling and drug research through the integration of stem cells [113]. In a recent study by Kemas et al. [83], it was found that PDMS exhibited a high absorption capacity for hydrophobic compounds. This characteristic could result in false negative findings during preclinical toxicity testing. For example, hepatotoxic compounds, such as chlorpromazine, might be incorrectly classified as safe when evaluated using PDMS devices [83]. In contrast, true toxic effects can be effectively identified with thiol-ene epoxy (TEE) devices, which demonstrate lower absorption rates. Consequently, it is advisable to consider low-absorption polymers such as TEE and polytetrafluoroethylene (PTFE) (polytetrafluoroethylene) for the development of BoC platforms. Furthermore, the application of coatings made from polyethylene glycol (PEG) or PTFE on the surface of PDMS can mitigate absorption rates. Considering these findings, there is a need to explore new material options for BoC systems and to investigate innovative polymers that not only possess low absorption characteristics but also meet the necessary optical and mechanical performance criteria.

## 5. Modeling Hematological Cancers in BoC

Cancer often possesses a complex microenvironment that features unique ECM, irregular vascularization, and limited perfusion. To accurately characterize a tumor tissue on a microfluidic platform, it is essential to incorporate cell cultures, appropriate growth factors, suitable media, and relevant readout components [114]. A BoC platform must be designed to integrate essential components, including bone, bone marrow, and immunological cells, to accurately represent hematological cancers. TME plays a critical role in the simulation of hematological cancers within BoC platforms. TME consists of both ECM components and a diverse range of cells that surround the tumor. This platform must also simulate the interactions among these cells as well as their relationship with the ECM. These cellular components are essential for protecting cancer cells from the immune system and enabling resistance to hypoxic conditions and pharmacological treatments [115]. TME, hematopoietic cells—including myeloid cells, T and B lymphocytes, NK cells, macrophages, and osteoclasts—coexist with non-hematopoietic cells such as stromal cells, MSCs, osteoblasts, osteocytes, fibroblasts, adipocytes, and endothelium [116]. Cell lines used to recapitulate the bone microenvironment are given in Table 6. MSCs are multipotent cells derived from the bone marrow, characterized by their ability to differentiate into various cell types [117]. Primary MSCs embedded in fibrin gel demonstrated the presence of smooth muscle and vascularization markers in a BoC model, which was developed using osteoblasts and GFP-labeled endothelial cells [88]. Furthermore, induced pluripotent stem cells (iPSCs) offer extended opportunities due to their ability to differentiate into a wide range of cell types [118]. Osteoblasts, which originate from MSCs, are integral to the formation of osteocytes. On the other hand, osteoclasts facilitate bone resorption, and their activity is modulated by OPG and RANKL secreted by osteoblasts [119]. Various cell types, such as mononuclear cells, leukocytes, and monocytes, have been employed in microfluidic chip platforms owing to their potential for osteoclast-like differentiation [120,121,122]. Paracrine factors released from osteocytes play a pivotal role in balancing the functions of osteoblasts and osteoclasts [41]. Osteoblasts, osteoclasts, and osteocytes function in a coordinated manner to sustain bone homeostasis [40]. This intricate collaboration effectively regulates the continuous processes of bone formation and resorption throughout an individual’s lifespan [123]. Stromal cells have been shown to reduce drug toxicity when included in co-culture and microfluidic chip studies, highlighting their supportive functions to other cell types [124,125]. Moreover, numerous studies explored primary stromal cells sourced from volunteer donors to examine their role in providing stromal support [14]. It is also essential to incorporate blood vessels and nerves within BoC systems, given their critical roles in the bone tissue [126,127]. Endothelial cells are essential for modeling bone tissue and promoting angiogenesis [128]. These cells also contribute to the secretion of growth factors and enhance the resistance to various pharmaceuticals [129,130]. Human umbilical vein endothelial cells (HUVECs) are commonly employed to model the vascularization process in BoC platforms [50]. To demonstrate vascularization on these platforms, HUVEC and MG63 cells were cultured on alginate microfibers, resulting in the formation of a double-layered osteon-like structure that resembled in vivo conditions [131]. In a different microfluidic system, bone, stromal, and endothelial cells were cultured in distinct compartments while still maintaining the interaction, which enhanced the endothelial cells’ capability to promote angiogenesis [87]. A recent study successfully simulated the vascular and nervous systems within the bone niche and demonstrated the ability to inhibit the neuronal growth associated with pain in inflammatory diseases through the use of nanoparticles [132]. Consequently, it is imperative to incorporate these cells into hematologic cancer microfluidic chip models specifically designed for drug research [133].

BoC platforms designed for modeling hematological cancers should exhibit a well-defined three-dimensional network of cells across various cultures and developmental stages while also demonstrating mechanical properties akin to those of natural bone tissue. The distinction between 2D and 3D cell cultures within microfluidic systems is a critical consideration. Related studies demonstrated that cells cultivated in 2D exhibited greater drug toxicity compared to those grown in 3D [134]. Furthermore, inconsistencies often arise between the drug toxicity in clinical studies and that in 2D cell cultures [135]. This difference is likely attributed to the lack of complex in vivo tissue architecture, intercellular communication, and interactions with the ECM in 2D environments [136]. Moreover, the integration of 3D cell cultures and stem cells into microfluidic chips allows for a more accurate representation of tissue and organ-specific metabolism, gene expression, and physiological processes [137]. Additionally, a related study demonstrated that 3D tumor spheroids cultivated on microfluidic devices were well-suited for drug screening applications [138]. The interactions between the tumor cells and ECM are instrumental in facilitating various cancer-related processes, including growth, proliferation, inflammation, immune suppression, angiogenesis, drug resistance, and metastasis [139]. ECM components —such as collagen, fibronectin, laminin, hyaluronic acid, and heparan sulfate— along with soluble factors, including cytokines, chemokines, and growth factors, significantly contribute to tumorigenesis [140]. Biomaterials play a vital role in providing sufficient mechanical support to cells by effectively mimicking the ECM [67]. Some ECM materials used in coatings to support the bone microenvironment are given in Table 4. It is essential to select suitable materials for the construction of artificial ECMs that are biocompatible, non-toxic, porous, and biodegradable [141]. The flexibility of the bone tissue matrix is primarily provided by type I collagen, whereas hydroxyapatite (HA) is responsible for bone stiffness [142]. Given that collagen alone cannot fully recapitulate the complexity of the bone and bone marrow microenvironment, the incorporation of hydrogels such as fibronectin and Matrigel is preferred in BoC platforms [143]. The integration of collagen with either fibronectin or Matrigel hydrogels effectively sustains numerous physiological cell functions. This strategic combination enhances cell viability and leads to a cell phenotype that is close to in vivo conditions [144]. Moreover, these hydrogels were shown to modulate cellular responses to various therapeutic approaches, including chemotherapy, immunotherapy, and radiation therapy [145]. Fibronectin, a critical element of the ECM, facilitates the essential communication between the intracellular and extracellular environments by binding to integrin receptors on cell surfaces, thereby influencing cell behavior [146]. Extensive research efforts demonstrated that fibronectin enhances cell adhesion and spreading while also affecting the cell migration pathways [147]. Matrigel is a gelatinous mixture that is comprised of proteins and growth factors secreted by Engelbreth–Holm–Swarm mouse sarcoma cells. It contains a variety of growth factors, including epidermal growth factor (EGF), fibroblast growth factor (FGF), nerve growth factor (NGF), platelet-derived growth factor (PDGF), insulin-like growth factor 1 (IGF-1), and transforming growth factor beta (TGF-β) [148]. In addition, HA provides a supportive environment for the proliferation of osteoblasts within BoC platforms and contributes to mechanical durability [149]. It plays a pivotal role in maintaining the balance of calcium and phosphate ions in the bone tissue, thereby being a key player in the bone regeneration process involving osteoblasts and osteoclasts [13]. To accurately simulate the characteristics of each hematological cancer within the in vitro BoC model, it is essential to incorporate the unique physical, mechanical, and biochemical properties associated with each pathology. Additionally, to effectively capture in vivo drug responses in BoC platforms, it is imperative to consider and integrate all these processes into the design of microfluidic chips.

BoC platforms developed to date primarily focused on bone metastasis in various solid tumors and processes of bone vascularization and remodeling [40]. One notable advancement was linked to a microfluidic system designed to investigate the impact of fibroblast-like synoviocyte migration and invasion on bone erosion, which was pertinent for both rheumatoid arthritis treatment and drug screening [150]. Additionally, a neurovascularized bone chip was utilized to further understand the mechanisms underlying osteoarthritis and evaluate the effects of various therapeutic agents [132]. Considering the bone’s relationship with the circulatory system, there have been considerable research efforts focusing on cancers that metastasize to the bone [151]. For instance, a microfluidic platform was employed to elucidate the role of CXCR2 and CXCL5 in breast cancer metastasis to bone [152]. Furthermore, a study examining the metastatic colonization of breast cancer within the perfused perivascular bone niche indicated that resistance to treatment could be associated with slow cell proliferation [50]. In the context of prostate cancer, co-cultured cells exhibited a more protrusive phenotype compared to the parental cell line, suggesting a potential link to reactive oxygen species [86].

**Figure 2:**
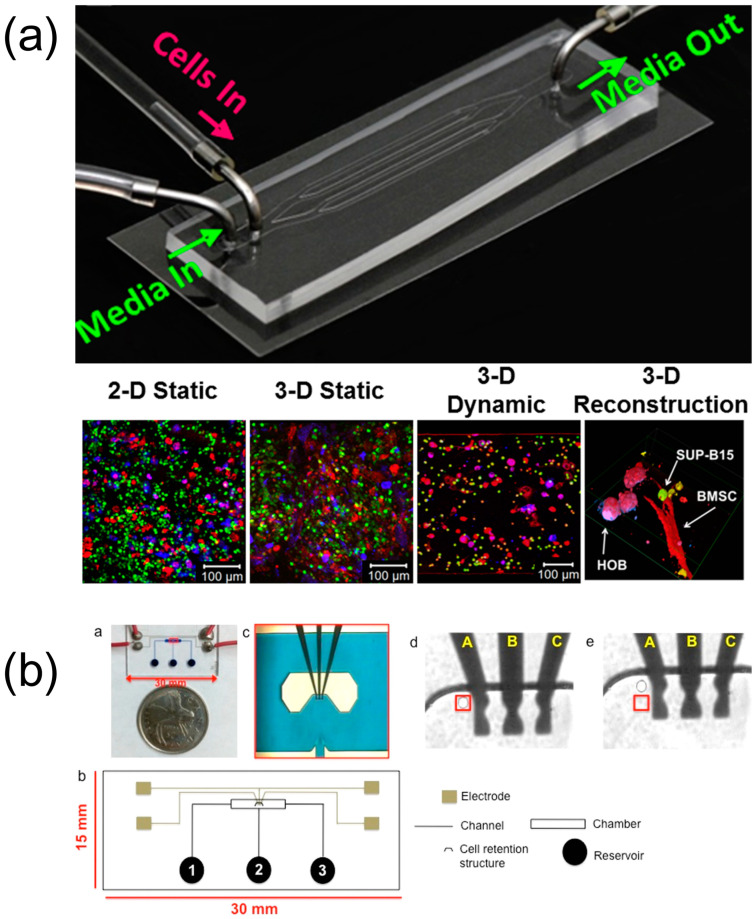

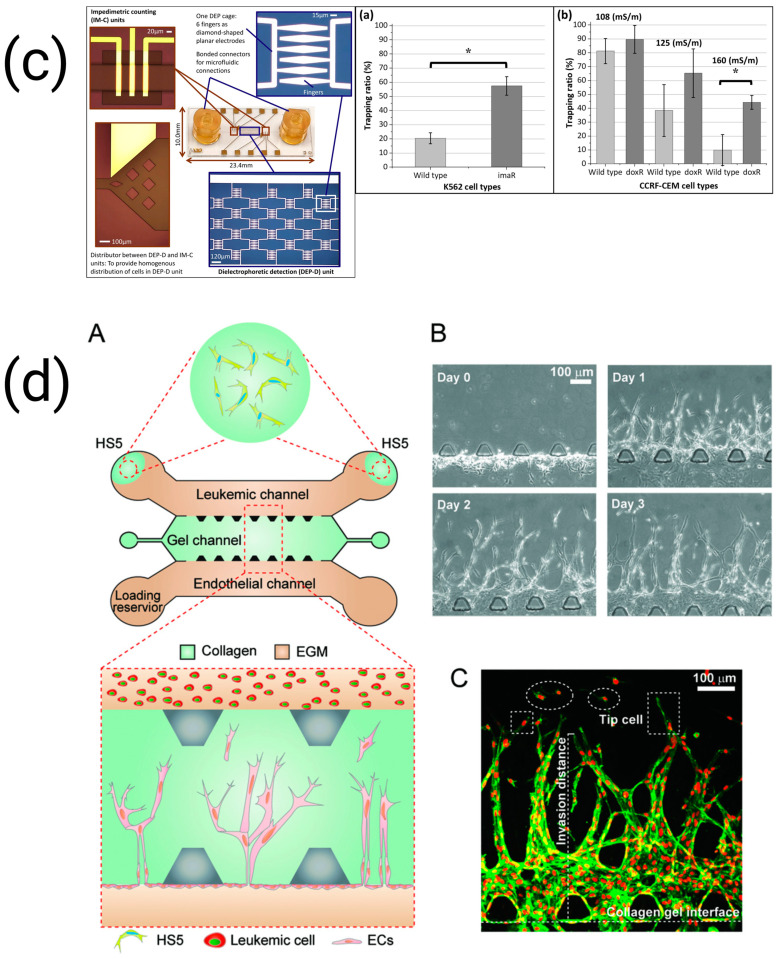
Related microfluidic chip studies on leukemia. (**a**) A platform consisting of 4 channels depicting the 3D bone marrow microenvironment embedded in collagen 1 is shown with SUP-B15 (green), osteoblasts (blue), and bone marrow stromal cells (red) [153], Copyright 2015, Plos One. (**b**) A dielectrophoretic microfluidic chip was developed to capture AML patient samples at the single-cell level and to measure MDR. (sub-a) Image of microchip filled with blue food dye. (sub-b) Layout of the microfluidic DEP chip, left reservoir (1) serves as cell inlet, middle reservoir (2) serves as drug delivery, and right reservoir (3) serves as waste. (sub-c) Close-up view of the compartment with DEP electrode. (sub-d,e) An image of a cell held near electrode A is shown in red square [155], Copyright 2016, American Chemical Society. (**c**) A microfluidic chip was developed by combining dielectrophoretic detection and impedimetric counting techniques and its effectiveness in selecting drug-resistant clones of K562 and CCRF-CEM. (sub-a) The trapping ratio of K562/wt and K562/imaR cells. (sub-b) The trapping ratio of CCRF-CEM/wt and CCRF-CEM/doxR cells in different buffers, with 108, 125, and 160 mS/m conductivity (*: *p* < 0.05) [156], Copyright 2021, Springer Nature. (**d**) (sub-A) Schematic of a 3-channel microfluidic system simulating leukemia cell-mediated angiogenesis. (sub-B) Phase-contrast images showing the invasion of endothelial cells in the collagen gel to form neovessels on days 0–3. (sub-C) Confocal image of endothelial cells germinating and migrating from the collagenous channel to the leukemic channel on day 3 [F-actin (green) and nuclei (red)]. The tip cells are indicated with dashed lines and the distance between them and the collagenous channel is shown as the invasion distance [157], Copyright 2016, Advanced Healthcare Materials.

The current efforts in exploring hematological cancers and the cytotoxic effects of drugs on BoC platforms are notably limited. The literature was comprehensively reviewed, with relevant studies elaborated upon in detail. Additionally, comprehensive strengths, weaknesses, opportunities, and threats (SWOT) analyses are presented in the related tables for clarity and ease of reference (Table 7, Table 8 and Table 9). A comparative analysis of the cytotoxic effects of the drug cytarabine across 2D static, 3D static, and 3D microfluidic cultures revealed that the 3D models exhibited reduced sensitivity to the drug (Figure 2) [153]. This innovative system facilitated an understanding of the interactions between cell-to-cell and cell-to-ECM models. In a study involving the TF-1 acute myeloid leukemia (AML) cell line and a stromal cell line, researchers cultured the cells using both conventional 2D methods and 3D microfluidic techniques, effectively characterizing the bone marrow niche [154]. They utilized scanning electron microscopy to compare cell morphologies across 2D and microfluidic cultures and assessed the cytotoxic effects of both azacitidine and cytarabine. Additionally, BCL2 expression levels were analyzed to determine the apoptosis rate through quantitative real-time polymerase chain reaction (qRT-PCR) [154]. Their results indicated that the 3D microfluidic system produced more normal cell morphologies and proliferation. Furthermore, drug resistance was found to be heightened in the 3D microfluidic cultures, with a correspondingly lower apoptosis rate compared to 2D cultures. The evaluation of daunorubicin resistance in AML patients was conducted through a dielectrophoretic chip-based assay, which allowed for single-cell level trapping (Figure 2) [155]. To assess multidrug resistance (MDR) in patients, this study employed inhibitors targeting key ABC transporters (ABCB1, ABCC1, and ABCG2) [155]. The findings revealed the presence of MDR+ cells in both pretherapy and relapse patient cases, suggesting that these cells might contribute to the mechanisms underlying relapse. A recent study introduced a microfluidic system that employed coupled dielectrophoretic detection and impedimetric counting to identify drug-resistant leukemia cells (Figure 2) [156]. This innovative system demonstrated the capability to differentiate imatinib-resistant cells from their wild-type counterparts, as well as to distinguish doxorubicin-resistant CCRF-CEM cells from their wild-type equivalents (Figure 2). Additionally, a biomimetic system was established to model leukemic cell-induced bone marrow angiogenesis, utilizing three different leukemia cell lines alongside endothelial and stromal cells (Figure 2) [157]. The secretions of angiogenic factors VEGF and bFGF in both monoculture and coculture were compared using qRT-PCR [157]. Those leukemic, endothelial, and stromal cells demonstrated increased angiogenesis during coculture, highlighting the critical role of cell-to-cell interactions. In another study, Aljitawi et al. investigated the drug responses of doxorubicin through the co-culturing of the HL60 leukemia cell line with stromal cells in both 2D and 3D configurations [158]. Accordingly, the cells in the 3D culture exhibited greater resistance to drug-induced apoptosis, and it was proposed that the different resistance observed in leukemia cell lines may be mediated by N-cadherin, which provided valuable insights into how various culture techniques could lead to different drug responses [158].

**Figure 3:**
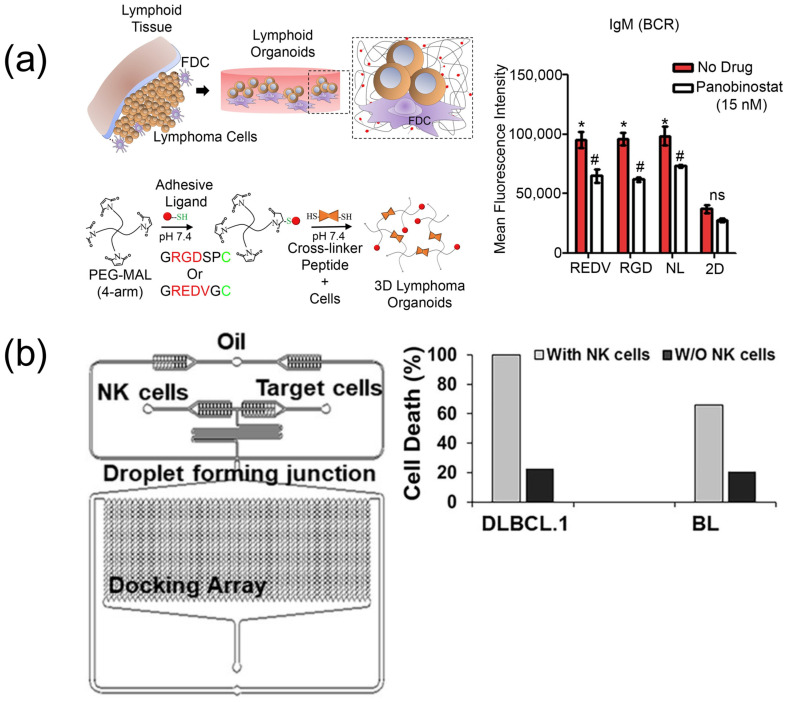

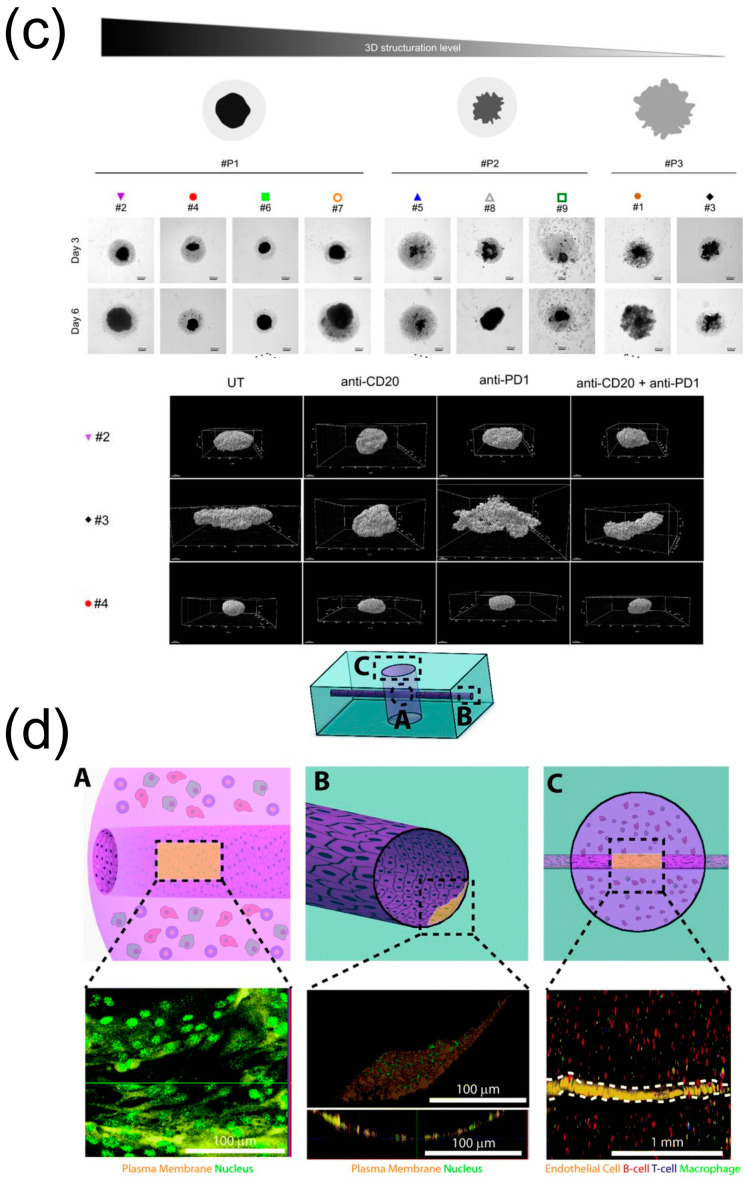
Related studies on the characterization of the lymphoma microenvironment. (**a**) Organoids were formed by encapsulating B- and T-cell lymphomas with follicular dendritic cells in a hydrogel, and the effect of Panobinostat on BCR expression was investigated (ns: non-significant, # and *: *p* < 0.05) [159], Copyright 2015, Biomaterials. (**b**) Droplet microfluidic platform developed for B-cell NHL and the effect of NK cells on patient samples [160], Copyright 2017, Frontiers in Immunology. (**c**) Nine FL patient samples with morphologies observed by bright-field microscopy and 3D reconstruction by IMARIS (880 confocal acquisitions at ×10 magnification) of treatment responses for patients 2, 3, and 4 [161], Copyright 2023, Journal for Immunotherapy of Cancer. (**d**) General schematic of DLBCL-on-a-chip and confocal micrographs from specific regions. MLMVECs were cultured in the DLBCL hydrogel in compartment A and the PDMS macrostructure in compartment B. The endothelial monolayer (white dotted line) in compartment C recapitulates the DLBCL tumor microenvironment by proliferating close to T cells, B cells, and macrophages within the DLBCL hydrogel [162], Copyright 2017, Lab on a Chip.

An investigation of the heterogeneous nature of lymphoma, particularly the emergence of malignant B and T cells and their impact on disease progression, utilized a DLBCL 3D organoid model (Figure 3) [159]. Within this 3D microenvironment, B cells were observed to secrete the B-cell receptor (BCR), resulting in enhanced cellular proliferation and increased resistance to the drug Panobinostat (Figure 3). This innovative system holds promise for facilitating long-term studies on hematological cancers and their response to various therapeutic agents. Moreover, a microfluidic gene expression array was developed to assess the patterns and cytotoxicity of NK cells in cases of B-cell NHL (Figure 3) [160]. The related findings suggest that lymphoma cells making a singular contact with primary NK cells exhibited accelerated cell death rates compared to those making multiple brief contacts. Notably, when evaluating the cytotoxicity of the NK-92 cell line within this microfluidic platform, it displayed greater effectiveness compared to primary NK cells. This microfluidic technology presents a significant advancement in enabling NK-based genetic analyses and cytotoxicity assessments at both clinical and single-cell levels (Figure 3). A novel system successfully cultured patient-derived Follicular lymphoma (FL) cells in a microfluidic environment, allowing for the formation of spheroids (Figure 3) [161]. Furthermore, the efficacy of obinutuzumab and nivolumab was evaluated within this framework, demonstrating a significant reduction in tumor burden (Figure 3). This device effectively simulated the pathological environment of FL with primary cells and presents a promising platform for the exploration of novel immunotherapeutic strategies. Additionally, a lymphoma–chip model incorporating immune, endothelial, and cancer cells was developed to simulate in vivo conditions pertinent to DLBCL (Figure 3) [162]. This system was characterized by a hydrogel-based circular microchannel consisting of readily accessible and cost-effective materials, thereby negating the requirement for advanced microfabrication techniques. The successful formation of microvessels on this chip indicates its potential as a suitable platform for drug research (Figure 3).

**Table 8:** SWOT analyses of Lymphoma BoCs.

Strengths	Weaknesses	Opportunities	Threats	Ref.
**Cell microenvironment adaptation:** Hydrogels provide a realistic tumor microenvironment for B and T cells.	**Manufacturing and scalability issue:** Manufacturing hydrogels for clinical use is complex.	**Use in immunotherapy testing:** Hydrogel-based systems can be used to evaluate the efficacy of T cell-based therapies.	**Regulatory uncertainty:** Lengthy validation processes are required for the clinical validity of such new platforms.	[159]
**Modularity and biomimetic properties:** Hydrogel-based platforms can be adapted to different cancer types.	**Lack of dynamism:** Tumor-immune cell interactions can be difficult to follow over the long term.	**Development of personalized models:** Patient-specific organoids can be created.	**Competition with alternative technologies:** May have to compete with other non-organoid 3D culture techniques.	
**Monitoring NK cell responses at the single-cell level:** Enabling more accurate immunotherapy assessments.	**Long analysis time per cell:** Measurements at the single-cell level require high time and resources.	**Optimization of next-generation immunotherapies:** Activation and inhibition of NK cells can be better understood.	**Data processing and analysis challenges:** Single-cell analyses requiring large datasets require bioinformatics infrastructure.	[160]
**Dynamic analysis with living tumor cells:** Immunological changes in lymphoma cells can be followed over time.	**Lack of standardization:** Further standardization of measurements is needed for clinical transition.	**High-resolution drug tests:** Individual lymphoma cells’ drug responses can be measured.	**Sensitivity issues:** NK cell interactions can be complex and misinterpreted.	
**Patient-derived spheroids:** Potential to accurately reflect the true tumor microenvironment.	**Scalability issue:** Patient-derived models may have limited use in large-scale drug testing.	**A powerful tool for personalized medicine:** Patient-based lymphoma models could make treatment choices more precise.	**Requirement for complex bioinformatics analysis:** Modeling patient-specific tumor-immune interactions requires big data analysis.	[161]
**Modeling the immune system’s effect on tumors:** Could provide better insight into lymphoma treatments.	**Generalizability to different patient groups:** Applicability to a broad patient population may be limited as the tumor microenvironment of each patient varies.	**Opportunity to understand immune-tumor interactions:** Provides the opportunity to better optimize immunotherapy strategies.	**Long validation processes:** More studies may be needed to put it into clinical use.	
**Modeling the tumor microenvironment with the vasculature:** Advantage of reflecting the real tumor environment more accurately.	**Technological complexity:** Microfluidic systems can be difficult to create and maintain.	**Testing new vascular-targeted therapies:** Could be used for angiogenesis inhibiting therapies.	**Expensive equipment requirements:** Microfluidic systems may require special laboratory equipment.	[162]
**Drug distribution and vascular permeability analysis:** The effects of new-generation drugs at the microvessel level can be tested.	**Low cell count compared to standard cultures:** May be limited in use in large-scale experiments.	**Modeling combination therapies:** May be useful in understanding how immunotherapy and vascular-targeting agents work together.	**Regulatory and clinical transition challenges:** Integrating models that are successful in the laboratory setting into clinical processes may take a long time.	

**Figure 4:**
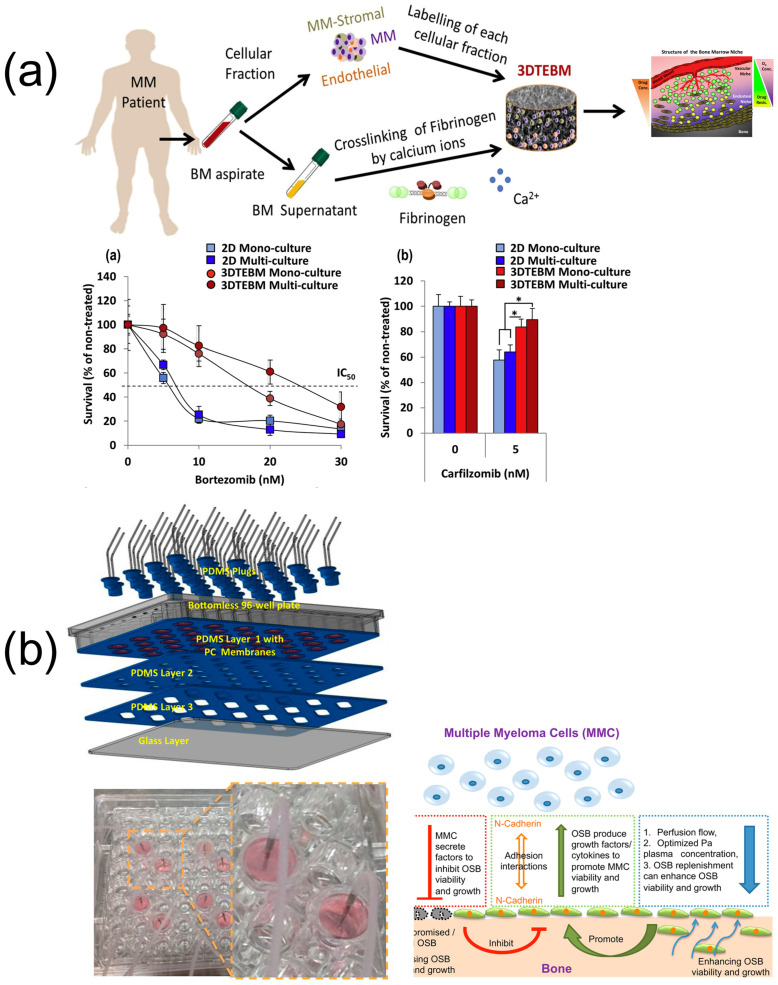

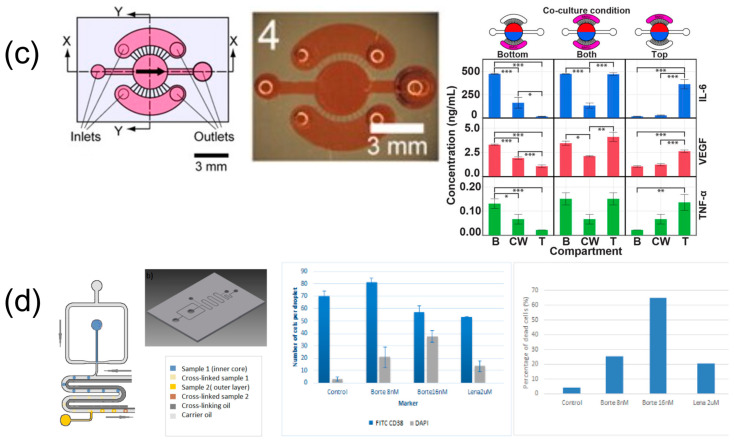
Related studies on MM microenvironment (**a**) Schematic of 3D cultured cells and bone marrow microenvironment. Survival rates of MM cells in 24 hours (sub-a) bortezomib and (sub-b) carfilzomib treatment in different culturing techniques (*: *p* < 0.05) [163], Copyright 2015, Biomaterials. (**b**) Schematic of the 96-well plate-based perfusion device and the interactions required for maintenance of MM samples from the patient [165], Copyright 2015, Plos One. (**c**) Microfluidic platform where MM and stromal cells communicate via diffusion channels and IL-6, VEGF, and TNF-α concentrations under different coculture conditions (*: *p* < 0.05, **: *p* < 0.01, ***: *p* < 0.001) [166,167], Copyright 2012, Blood and Copyright 2016, Biomicrofluidics. (**d**) Schematic of the double-layered hydrogel bead generator and therapeutic effect of bortezomib and lenalidomide in patient-derived MM cells [168], Copyright 2020, Micromachines.

A three-dimensional model was proposed to generate oxygen and drug gradients, thereby effectively illustrating the interactions among MM cells, stromal cells, and endothelial cells in bone marrow (Figure 4) [163]. This advanced system provides valuable insights into drug resistance, significantly contributing to our understanding of MM pathophysiology (Figure 4). A hexagonal platform was developed to investigate the interactions among osteoblasts, primary MM cells, and bone marrow mononuclear cells [164]. This system incorporates a patient-derived plasma medium, enabling the primary MM cells to sustain their viability for three weeks. However, the characteristics of the MM disease hindered the development of osteoblasts within this model. Consequently, a high-throughput well plate-based perfusion device, which facilitated the extension of primary MM cell viability through the renewal of perfusion flow, plasma, and osteoblasts, was fabricated (Figure 4) [165]. A PDMS device was developed for the co-culture of the RPMI8226 multiple myeloma cell line in conjunction with the Hs.5 stromal cell line using perfusion channels (Figure 4) [166]. The response of the MM cells to Bortezomib was assessed using this device, which showed notable translocations of NF-κB and STAT3 in the cells. Additionally, a primary sample from a chronic lymphocytic leukemia patient was analyzed to validate this chip’s efficacy, and it demonstrated significant NF-κB activation [166].

**Table 9:** SWOT analyses of Myeloma BoCs.

Strengths	Weaknesses	Opportunities	Threats	Ref.
**Realistic microenvironment modeling:** 3D engineering of bone marrow may more accurately reflect the pathophysiology of myeloma.	**Technological challenges:** Standardization of 3D tissue engineering techniques is complex.	**A powerful model for personalized medicine:** Patient-specific bone marrow environments can be generated.	**Competition with alternative models:** May be more complex compared to organoids or other in vitro systems.	[163]
**Suitable for drug resistance studies:** How myeloma cells respond to drugs in the bone marrow microenvironment can be studied.	**Scalability issues:** Large-scale production suitable for clinical use may be difficult.	**Potential for developing immunotherapy:** Better understanding of how myeloma cells interact with the immune response.	**Regulatory approval processes:** May require lengthy validation for clinical application.	
**Realistic osteoblastic niche modeling:** Allows myeloma cells to survive longer in the bone marrow environment.	**Reproducibility issue:** There may be biological variations among patient samples.	**Personalized drug testing:** Individualized treatments can be developed with patient-derived myeloma cells.	**Limitations compared to in vivo models:** The extent to which the ex vivo environment reflects whole-body physiology is questionable.	[165]
**Long-term maintenance of primary cells obtained from patients:** Provides the opportunity to analyze clinical samples for a long time in a laboratory environment.	**The cell culture process is complex:** It requires specific optimization for long-term primary cell maintenance.	**Developing targeted therapies:** New targets can be discovered by investigating osteoblast-myeloma interactions.	**High cost:** Primary cell maintenance and niche optimization can be costly.	
**Sensitive analysis with microfluidic systems:** Myeloma cells can be studied in a dynamic diffusion environment.	**Limitations of static systems:** Microfluidic systems may not fully reflect dynamic in vivo conditions.	**Ideal for advanced drug testing:** Provides a sensitive platform for testing new drug candidates.	**Challenges in large-scale testing:** Microfluidic systems may be limited for high-volume clinical testing.	[167]
**Functional analysis at the single-cell level:** Drug resistance and cell heterogeneity can be measured in detail.	**Costly technology:** Microfluidic chips can be expensive to manufacture.	**Analysis of combination therapies:** Provides a convenient model for studying the combined effects of different drugs.	**Difficulty of adaptation compared to more traditional methods:** There may be a learning curve depending on the methods researchers are accustomed to.	
**Longer survival of primary myeloma cells:** Myeloma cells can be preserved for long periods of time in an ex vivo environment.	**Technological infrastructure requirement:** Microdroplet systems require special equipment.	**A suitable model for precision medicine:** It may be possible to develop personalized treatments with patient-based myeloma cell cultures.	**Regulatory uncertainty:** Microfluidics-based cell cultures may not be sufficiently validated for clinical applications.	[168]
**High-throughput cell culture with microdroplet systems:** Provides small-volume but efficient cell growth.	**May not fully reflect heterogeneity:** It may be difficult to grow all patient tumor cells equally.	**Testing platform for immunotherapies:** Microfluidic systems can be used to analyze immunotherapy efficacy.	**Competition with alternative methods:** May be difficult to adapt compared to traditional cell culture systems.	

The co-cultured cells engaged in paracrine interactions through the secretion of cytokines, including IL-6, VEGF, and TNF-α (Figure 4) [167]. Moreover, STAT3 nuclear translocation was characterized at a single-cell level [167]. This device was also utilized for microfluidic co-culture (MicroC^3^) of the CD138 MM cells, both negative and positive, derived from a patient, and the responses of the patient cells to Bortezomib treatment were evaluated within this system [169]. This developed platform revealed the potential of diagnostic testing; however, challenges related to the proliferation and storage of primary MM cells constrained further stages of testing. Recently, a droplet microfluidic platform was presented to establish a three-dimensional biomimetic environment that closely resembled in vivo conditions for patient-derived MM cells and MSCs (Figure 4) [168]. Notably, the patient-derived MM cells demonstrated the ability to survive independently, without the presence of MSCs within the device. Furthermore, the MM cells within the droplets exhibited a continuous response to therapeutic agents (Figure 4). This microfluidic platform could be regarded as a promising platform for evaluating drug cytotoxicity in an ex vivo context.

## 6. Drug Testing and Imaging in BoC Platforms

In the context of disease modeling and drug research with the use of BoC platforms, cellular interactions with one another and ECM lead to the production and degradation of various biomolecules. Traditional 2D cell cultures often fail to accurately represent the drug efficacy; in contrast, 3D cell cultures provide a more realistic model, closely resembling the in vivo microenvironment. Biomolecules, such as mRNA, proteins, lipids, and various ions, serve as important biomarkers that provide critical insights into the disease status [62]. The integration of microfluidic platforms into drug research, testing, and their development not only improves the success rates but also reduces the associated costs and shortens the timeline for obtaining results [170]. Moreover, biosensors can be effectively combined into microfluidic chips to detect disease patterns and drug responses within BoC platforms. For instance, sensors capable of detecting metabolites such as glucose and lactate, along with important substances like oxygen and calcium, can be seamlessly incorporated into the platform [65]. This integration enhances the platform’s ability to explain biological processes and therapeutic responses. The identification of disease biomarkers through biosensors in BoC platforms primarily involves in vitro diagnostic tests. In this process, samples are obtained from cancerous tissue via conventional biopsy or liquid biopsy methods. A range of molecular techniques is utilized to detect hematologic cancer biomarkers, including immunohistochemistry, enzyme-linked immunosorbent assay (ELISA), electrophoretic mobility shift assays (EMSA), fluorescence in situ hybridization (FISH), flow cytometry immunophenotyping, reverse transcription polymerase chain reaction (RT-PCR), and chromosome analysis [171]. It is important to note that many of the current testing methods necessitate the disassembly of the chip and fragmentation of the disease model for use in BoC platforms. Biosensors emerge as the most appropriate tools for conducting analysis and detection directly on the chip, preserving the integrity of the disease model. Furthermore, sensors can be employed for high-throughput screening of analytes within a range of devices [172]. Low signal-to-noise ratios in microfluidic platforms might significantly compromise the sensitivity of biosensors, especially in dynamic environments [173]. The challenge of detecting low concentrations of biomolecules released from cells underscores the necessity for advanced detection techniques. Moreover, surface coatings and electrode materials utilized in biosensors must demonstrate compatibility with the cell culture medium [174]. In order to achieve biocompatibility, surface modifications may be necessary, particularly for metal electrodes such as gold and platinum, as well as for polymer-based sensors [175]. However, it is important to note that these modifications have the potential to affect the sensor performance or contribute to the deterioration over extended periods of use. The incorporation of biosensors into BoC systems can significantly elevate manufacturing costs. This challenge is especially pronounced in complex systems that require multiple sensors, which may lead to increased expenses for electronic components and data acquisition devices. Additionally, the transition from laboratory prototypes to industrial-scale production can pose challenges related to cost-effectiveness. It is important to consider cost-effective options as well [176]. The implementation of flexible and conductive polymers, coupled with the advancement of microelectromechanical systems (MEMS)-based sensors, has the potential to significantly enhance the functionality of BoC platforms in clinical and pharmaceutical applications [177].

Rapid readout and processing can be achieved by integrating chips with various testing units and incorporating a range of analytical systems capable of displaying diverse datasets within the overall framework. A range of sensors and detectors is incorporated within the system for drug imaging studies on microfluidic devices, offering advantages in integration, sensitivity, and rapid analysis. The methodologies for detection include fluorescence, Raman spectroscopy, mass spectrometry, and electrochemical techniques, with fluorescence being the most prominent and well-established method for monitoring cellular behavior and drug responses [178,179,180]. For example, in a recent study, P450 enzymes were analyzed via fluorescence to investigate the drug activity in liver cancer spheroids [181]. Additionally, another investigation employed Hoechst 33342/PI dye in conjunction with fluorescence microscopy to evaluate responses to various compounds [182]. A sandwich-type microfluidic chip platform facilitated the detection of breast cancer drugs using fluorescence imaging techniques [183]. Changes in the morphology of fluorescently labeled cells cultured in microfluidic chips can be effectively monitored using fluorescence microscopy. Additionally, laser-induced fluorescence analysis technology plays a crucial role in evaluating drugs’ pharmacological activities by assessing their effects on cells [76]. An innovative system featured a microvalve that enabled the creation of irinotecan gradients in hepatocellular carcinoma cells cultured on a microfluidic chip, thereby allowing for the examination of the drug’s effects at six distinct concentrations [184]. A microfluidic platform employing electrochemical detection methods was designed for the rapid screening of various compound concentrations on colon cancer cells [185]. A microfluidic system developed for simulating drug metabolism in the liver and evaluating cytotoxicity was successfully integrated with mass spectrometry [186]. Raman spectroscopy and mass spectrometry are valuable analytical techniques utilized for the detection of small molecules. Raman spectroscopy, along with surface-enhanced Raman spectroscopy, employs light scattering methods to effectively identify analytes [187], while mass spectrometry is a rapid technique that provides critical information regarding the molecular weight of the analyte [188].

Research efforts related to drug interactions with BoC platforms are limited. However, below is a summary of several studies related to bone tissue that may provide useful insights. In a related study, multiple channels were strategically arranged in a Christmas tree configuration on a microfluidic chip constructed from PDMS and hyaluronic acid materials [70]. This design facilitated the creation of a concentration gradient for drug administration, demonstrating considerable promise for the imaging of bone-related pharmacological agents. A microchamber made of PDMS and equipped with independent perfusion microchannels and an arrangement of cell chambers (measuring five by eight) could enable the simultaneous testing of seven anticancer drugs [189]. An engineered bone marrow device, developed with samples obtained from an implanted device in mice and demineralized bone powder, modeled hematopoiesis, as well as drug responses and toxicities in the bone marrow [190]. This innovation presents a valuable platform for advancing drug research in hematological diseases.

As a new approach, image analysis of BoC platforms could be supported by artificial intelligence (AI). A recent study highlighted the co-culture of IDG-SW3 and MC3T3-E1 cell lines within a well plate based on a three-dimensional gel unit [18]. This platform allowed for rapid and efficient drug testing, owing to its bone modeling and transparency. Antibody drugs designed for the treatment of osteoporosis were tested on this platform, revealing the potential for the development of bone models and drugs targeting bone-related diseases [18]. In a recent study, various small compounds were tested on vascularized microtumor tissues cultivated in 96-well plates, demonstrating the platform’s effectiveness in evaluating drug efficacy across diverse tissue types [191]. Additionally, a chip capable of multi-organ integration was designed to operate without pumps and in serum-free media, focused on the analysis of anti-leukemic drugs, and revealed significant cytotoxic effects of diclofenac and imatinib [192].

**Figure 5:**
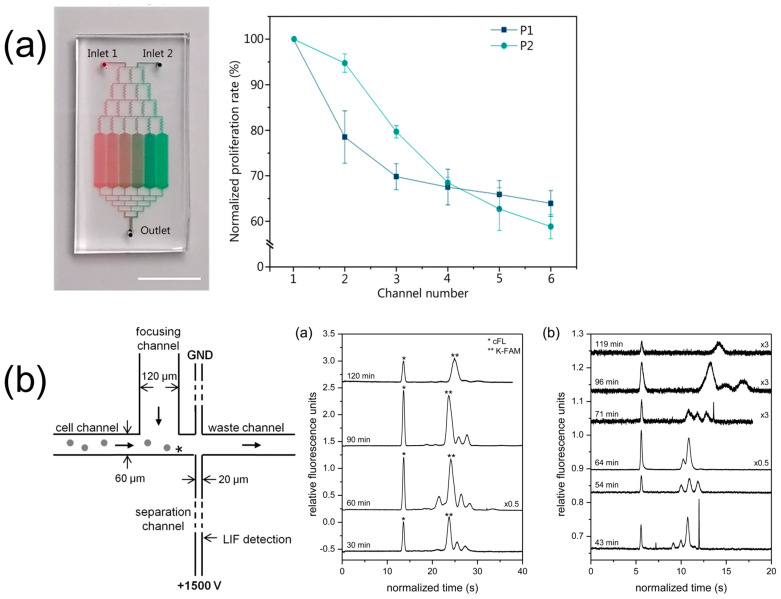

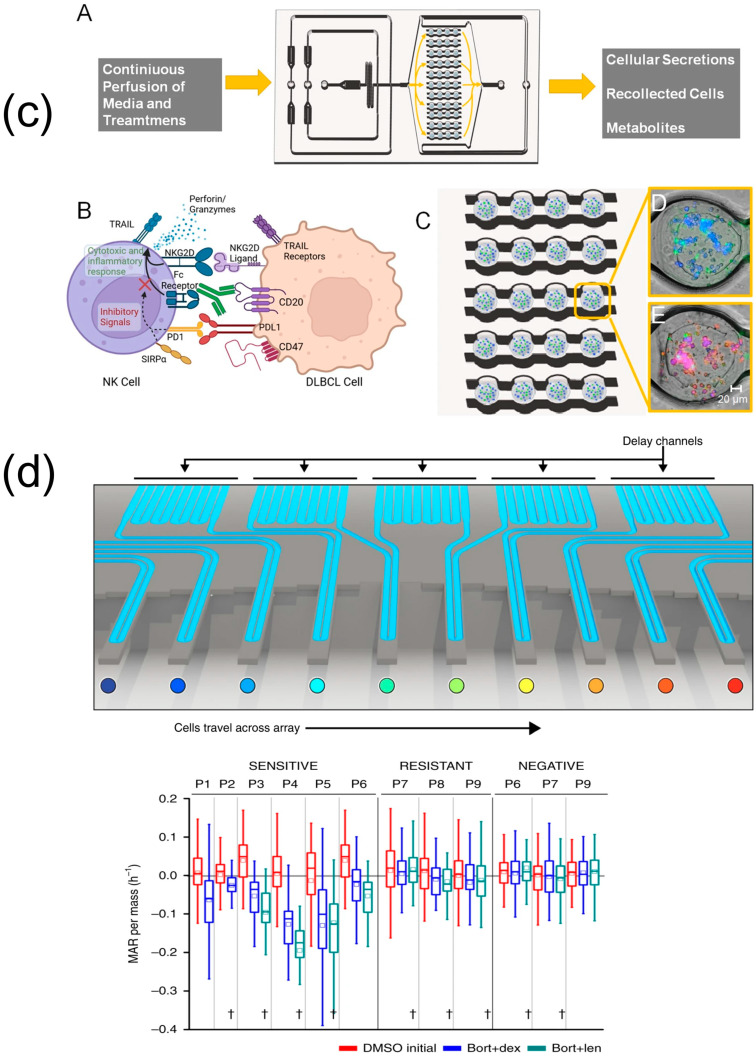
Related microfluidic chip platforms for investigating drugs used in hematological cancers (**a**) Image of the device colored with red and green dyes and the effect of drugs combined at 6 different concentrations with samples from two AML patients [194], Copyright 2022, Military Medical Research. (**b**) Schematic of the microfluidic device (* is the location of cell lysis) and electropherogram analysis of lysed AML cells (sub-a) before and (sub-b) after the drug administration [195], Copyright 2013, Analytical Chemistry. (**c**) (sub-A) Schematic of the microfluidic device and (sub-B) interaction of NK and DBLCL cells within the device. (sub-C–E) Status of DLBCL (blue), NK cells (green), and dead cells (red) within the device before and after rCHOP treatment [196], Copyright 2024, Cell Death and Disease. (**d**) The microfluidic platform, where the MARs of each MM cell were measured serially (the dots changing from blue to red correspond to the mass of the same cell measured consecutively with 10 different sensors), and the samples taken from patients were divided into sensitive, resistant, and negative groups, and the effects of different drugs and drug combinations were shown (†: denotes blinded tests) [198], Copyright 2017, Nature Communications.

BoCs can serve a crucial role in drug development studies, facilitating the assessment of potential side effects associated with candidate drugs [193]. Moreover, in the context of personalized therapy, BoC platforms can be utilized to evaluate the patient’s response to medications, utilizing cells obtained from the individual. Unfortunately, only a few BoCs were developed specifically for hematological cancer drug screening. Detailed analyses of strengths, weaknesses, opportunities, and threats (SWOT) are shown in Table 10. For instance, a specialized microfluidic cell culture chamber array was fabricated, known as Simple, Massive, Multiplex, Alive, Retainable, and Trackable (SMART) (Figure 5) [194]. This system facilitated the production of multiplex drug concentrations and was utilized to assess the responses of primary patient samples to daunorubicin and cytarabine (Figure 5). A microfluidic platform was developed to measure peptide degradation rates at the single-cell level through the application of chemical cytometry (Figure 5) [195]. This system assessed the efficacy of tosedostat treatment in the U937 cell line (Figure 5). A recent study introduced a microfluidic chip model utilizing hydrogel spheres to screen primary DLBCL cells for their interactions with NK cells and the rCHOP chemotherapy regimen (Figure 5) [196]. This system demonstrated capabilities in assessing cell viability, identifying surface markers, and evaluating changes in secretory profiles and gene expression (Figure 5). Consequently, it allowed for the comparison of various treatment approaches in lymphoma cells, facilitating advances in therapeutic strategies. A quadratic phenotypic optimization platform (QPOP) was presented to assess patient-specific drug combinations using biopsy samples from individuals diagnosed with NHL [197]. Accordingly, among the 12 drugs evaluated, the combinations involving copanlisib and romidepsin emerged as the most effective ones. Additionally, QPOP demonstrated a reduction in disease progression when compared to conventional chemotherapy in previously treated patient groups, which demonstrated that drug testing could be performed on ex vivo samples within a short time frame. A microfluidic platform known as a suspended microchannel resonator evaluated the ex vivo drug sensitivity in MM, utilizing the mass accumulation ratio (MAR) as a key metric (Figure 5) [198]. This system was employed to assess the responses of both MM cell lines and patient samples to standard chemotherapy agents, including dexamethasone, lenalidomide, and bortezomib, as well as to peptide-based therapeutic (Figure 5). When administered individually, these drugs resulted in a decrease in the MAR; conversely, when implemented as a combination therapy, the MAR exhibited an even more significant reduction. The efficacy of this platform lies in its ability to facilitate drug evaluation without the requirement for any extended ex vivo cultivation of MM cells.

## 7. Future Perspectives: The Capacity in Commercialization and Interpretation of Clinical Data

The increasing variety of human diseases and the rise of resistance to existing medications underscore the critical need for the urgent development of new therapeutic drugs. However, to bring a new drug to clinical use, significant time and financial investments are necessary. For instance, the process of obtaining regulatory approval and commercializing a newly discovered drug can last up to 15 years and incur a cost of approximately USD 2.8 billion [5,67,199]. Throughout this process, comprehensive evaluations of the safety, efficacy, toxicity, and pharmacokinetics of new compounds are essential [67]. Traditionally, the efficacy of new drugs is assessed using animal models prior to clinical trials in humans. However, it is important to recognize that these animal models may not accurately predict therapeutic effectiveness and potential side effects in humans due to inherent physiological and metabolic differences between species [200]. In this context, it is imperative to adhere to the internationally accepted principles of reduction, refinement, and replacement during research activities involving animal models [201]. In the pursuit of developing personalized treatment combinations and identifying innovative therapeutic compounds, there is a pressing need for advanced predictive tools. These systems should effectively guide disease management while mitigating the risk of resistance and yielding comprehensive insights from a reduced number of samples. The microfluidic chip platforms, known as Companion Diagnostics (CDx) (U.S. Food and Drug Administration, 2016), were designed to precisely identify the most suitable treatment strategies for individual patients [202]. CDx devices have several significant advantages: (1) They enable the identification of patient groups that are likely to benefit from specific medications; (2) They allow for the assessment of potential side effects associated with drugs, identifying high-risk patients who might be adversely impacted; (3) They facilitate the monitoring of drug responses, providing opportunities for toxicity assessment and dosage adjustments [202,203]; (4) CDx devices can expedite the drug development process and contribute to cost reduction, ultimately advancing the field of personalized medicine [6,204].

Many startups and established companies have been actively working to advance the commercial availability of OoC platforms. For instance, Hesperos is an advanced multi-organ-on-chip system designed to replicate the intricate interactions among muscles, tissues, nerves, and organs [205]. Additionally, it provides valuable information about the dynamics of drug metabolism, including processes of absorption, distribution, and excretion [205]. TissUse introduced HUMIMIC chips, a multiorgan platform capable of facilitating media perfusion without requiring a pump [206]. Mimetas developed OrganoPlate, which incorporates an innovative microfluidic channel system featuring hydrogel–liquid interfaces [207]. Additionally, the Phaseguides technology enables the cultivation of 2D and 3D cell cultures without barriers [208]. Netri is an OoC system that seamlessly combines neural stem cells with sophisticated AI algorithms [209]. Emulate generated a liver chip designed to economically assess the efficacy of various drugs [210]. Moreover, AIM Biotech developed a model to examine the impact of small molecules and engineered T-cell cytotoxicity on hepatocellular carcinoma [211].

The use of OoC systems in drug screening is a developing area, and several strategic collaborations have recently been formed. TissUse, for example, is partnering with AstraZeneca [212], while Emulate partnered with Johnson & Johnson (NJ, USA) [104]. The number of studies utilizing multi-organ on-chip systems to evaluate the effects of pharmaceuticals across various organs has been steadily increasing [213]. For instance, such a system was developed to examine the pharmacokinetic activity of cisplatin in bone marrow, liver, and kidney [214]. Furthermore, another multi-organ on-chip model, which simulated the immune system, was employed to study the cardiac, skeletal, and hepatic responses to amiodarone through the mechanism of monocyte actuation [215]. Moreover, AI has significantly enhanced drug imaging by improving accuracy, efficiency, and predictive capabilities. Deep learning algorithms enable automated image analysis, facilitating the identification of molecular interactions and structural changes in drug compounds. AI-driven imaging techniques, such as machine learning-based segmentation and pattern recognition, allow for precise visualization of drug distribution at the cellular and tissue levels. Moreover, AI-powered image processing accelerates high-throughput screening and drug discovery by identifying potential therapeutic targets with greater speed and reliability [216,217]. As AI continues to evolve, its integration into drug imaging promises more efficient and personalized approaches in pharmaceutical research and development.

To achieve acceptance of BoC platforms in clinical and commercial applications, these innovations need to undergo some approval processes outlined by regulatory bodies such as the US Food and Drug Administration (FDA) and the European Medicines Agency (EMA). Current challenges include the absence of clear and standardized guidelines from these organizations regarding innovative technologies such as BoC systems, which can impede the commercialization timeline. Specifically, extensive validation studies and multi-center trials may be necessary to demonstrate the clinical predictive accuracy of in vitro models. Furthermore, the diverse production standards employed by various research groups and manufacturers for components such as microfluidic devices, biomaterials, and bio-sensor integrations further complicate the reliable reproducibility of results. This lack of standardization represents a significant barrier against the acceptance of BoC systems as trustworthy tools within the pharmaceutical industry. To address this issue, organizations such as the International Organization for Standardization (ISO) and the American Society for Testing and Materials (ASTM) must focus on developing more comprehensive standards in this field. Currently, BoC platforms utilized in research laboratories typically rely on small-scale and manual production processes. However, for their successful implementation in pharmaceutical and clinical settings, high-volume and cost-effective production solutions are required. The integration of microfluidic devices into injection molding, along with automated production lines and packaging processes, presents potential scalability solutions. Furthermore, BoC systems need to adhere to critical requirements such as sterility, biocompatibility, and extended shelf-life factors that are vital for mass production processes. To enhance the commercialization potential of BoC systems, it is imperative to expedite validation and standardization efforts in collaboration with regulatory institutions. Additionally, there is a need to develop suitable materials and design solutions tailored for large-scale production. Toward this end, the fabrication of reliable and cost-effective prototypes suitable for clinical studies will be essential for the acceptance of BoC platforms within the medical and biotechnology sectors. As a result, it is within our reach to envision the creation of BoC platforms that will empower clinical translation and personalized therapies, driving groundbreaking research in the field of hematological cancers.

## 8. Conclusions

BoC platforms trigger a valuable advancement in biomedical technology, particularly for the modeling of biological processes associated with the bone. These platforms hold significant potential for applications in bone regeneration, disease modeling, drug testing, and translational medicine. For personalized therapeutic approaches in hematological cancers and preclinical evaluation of potential drug candidates, BoC platforms provide a significant reduction in both cost and time. Despite this potential, platforms designed for hematological cancers and high-throughput drug testing using the BoC technology were not frequently reported in the literature. To enhance the integration of BoC devices into CDx platforms, it is essential to incorporate diverse arrays of biosensors. This integration will certainly facilitate the functionality and broaden the utility of these devices in clinical applications. Furthermore, the incorporation of optical lenses with distinct properties, along with sensors capable of accurately measuring various electrochemical properties, into microfluidic chips will significantly improve our scientific capabilities. Moreover, the application of algorithms such as AI, deep learning, and machine learning will streamline the processing and visualization of data generated by these biosensors, ultimately resulting in more efficient and precise testing. While the integration of high-throughput screening techniques into BoC platforms presents significant opportunities, it is important to recognize several existing limitations. Notably, the costs associated with robotic attachments necessary for liquid handling and data collection can be substantial. Additionally, the expenses related to data libraries for drug screening and the materials required for cell studies pose challenges that may impede the continuity of research efforts. Strengthening the collaboration among universities, clinics, and companies and supporting these interdisciplinary research efforts with generous grants will be crucial in advancing these initiatives.

## Figures and Tables

**Table 1:** BoC development milestones.

Key Development	Technology Innovation	Year	Reference
Introducing the first BoC platforms	Combination of microfluidic technologies and cell culture	2010	[15]
Development of bone marrow-on-a-chip system	In vitro blood cell production and modeling of the bone marrow microenvironment	2013	[16]
Design and evaluation of an osteogenesis-on-a-chip device	Integration of 3D microenvironment and fluid shear stresses	2020	[12]
Designing hydrogel-based BoCs for personalized medicine	Use of hydrogel to provide 3D culture environment for cells	2021	[17]
Combining high-throughput biomimetic BoC platform with AI-based image analysis	Artificial intelligence-assisted image analysis and osteocyte-osteoblast coculture	2023	[18]

**Table 2:** An outline of the review on BoCs for hematologic cancers.

Section	Description
**The biological landscape of hematologic cancers**	Overview of hematologic cancers, including leukemia, lymphoma, and multiple myeloma, and their clinical diagnosis and treatment.
**Simulating bone in microfluidic chip platforms**	Techniques for replicating bone physiology using microfluidic systems, focusing on the bone marrow niche and cell–cell interactions.
**Materials and manufacturing processes for fabrication of BoC platforms**	Comparison of materials (e.g., PDMS, PMMA, hydrogels) and manufacturing methods (e.g., soft lithography, 3D printing) used in BoC systems.
**Modeling hematological cancers in BoC**	Application of BoC models in studying specific hematological cancers, including disease progression and treatment response.
**Drug testing and imaging in BoC platforms**	Methods for assessing drug efficacy and toxicity within BoC systems, incorporating imaging techniques and biosensors for real-time analysis.
**The capacity for commercialization and interpretation of clinical data**	Exploration of the challenges and opportunities for translating BoC technologies to clinical and pharmaceutical markets, including regulatory considerations.

**Table 3:** Chemotherapeutic drugs used in hematological cancers.

Hematologic Cancer	Drugs
ALL	daunorubicin, vincristine, prednisone, and asparaginase
CLL	ibrutinib, zanubrutinib, acalabrutinib, obinutuzumab, and rituximab
AML	cytarabine, daunorubicin, and idarubicin
CML	imatinib, dasatinib, and nilotinib
HL	doxorubicin, bleomycin, vinblastine, and dacarbazine
NHL	cyclophosphamide, doxorubicin, vincristine, prednisone, and ± rituximab
MM	bortezomib, lenalidomide, and dexamethasone

**Table 4:** Some indications of bone microenvironment delineation in microfluidic chips.

Indicator	Method	Biomarker	Reference
Osteoblast, osteoclast, and osteocyte interactions	Immunohistochemistry	Osteocalcin, Col1, and BSP	[70,71]
	Fluo-4, Alizarin red, and von Kossa dyes	Calcium	[12,55,72]
	ELISA	OPG and RANKL	[52]
	Enzymatic reactions	ALP and TRAP	[62]
	Gene expression	RUNX2, ALP, Col1, BSP2, osteocalcin, TRAP, CTSK, MMP-9, RANK, OPG, and RANKL	[18,70]
Cell morphology	DAPI and Hoechst 33342 etc.	Nuclear shape index	[73]
Cell secretions	ELISA	TNF-α and IL-6	[74]
Cell metabolites	Nile Red and BODIPY derivatives	Lipids	[75]
Vascularization	Gene expression	VEGFR1 and VEGFR2	[50]

**Table 5:** Some materials used in fabrication and coatings to mimic bone microenvironment and ECM.

Main Purpose	Materials	Reference
Bone compartments	PDMS	[85]
Bone biomimetics	PDMS-Glass	[18]
Bone microenvironment and ECM	PDMS-Glass coverslip/Fibronectin-type I collagen	[86]
	PDMS-Glass/Hydroxyapatite -Fibrin	[87]
Bone microenvironment	PMMA-PDMS-Glass	[52]
Bone microenvironments	Polymerized high internal phase emulsion scaffold-PDMS-Glass	[12]
Vascularized bone microenvironment	PDMS-Glass/Fibrin	[88]
Bone perivascular niche	Polycarbonate-PDMS-Glass/Decellularized calf metacarpal joint	[50]

**Table 6:** Major cell lines used to simulate the bone microenvironment.

Cell Name	Cell Code
Human bone marrow-derived mesenchymal stem cells	PCS-500-012
Human umbilical cord-derived mesenchymal stem cells	PCS-500-010
Murine osteocyte-like cell line	MLO-Y4
Mouse macrophage cell line	RAW 264.7
Mouse preosteoblast	MC3T3-E1
Human osteoblast-like cells	MG63
Human osteoblast cell line	hFOB 1.19
Human bone marrow stromal cell line	HS-5, HS-27A
Mouse fibroblast cell line	3T3 cells
Human fibroblast cell line	SW-1353
Human umbilical vein endothelial cell line	HUVEC
Rat umbilical vein endothelial cells	RUVEC
Human neuronal cells	SH-SY5Y

**Table 7:** SWOT analyses of Leukemia BoCs.

Strengths	Weaknesses	Opportunities	Threats	Ref.
**3D culture system:** Ability to model the bone marrow microenvironment in three dimensions.	**Model complexity:** A complex environment such as bone marrow can be difficult to fully simulate.	**Innovative treatment models:** Can be a powerful tool for high-throughput treatment testing.	**Dominance of classical cell cultures:** Traditional 2D cell cultures are still widely used.	[153]
**Biomimetic features:** Simulation of interactions in the microenvironment of leukemia cells.	**Implementation limitations:** Translating the model into clinical applications may take time.	**Targeted therapy and drug testing:** May be useful in developing individual treatment approaches.	**Financial and logistical barriers:** Developing microfluidic devices can be costly.	
**Targeted therapy research:** Opportunity to directly study the effectiveness of cancer treatments.	**Technological requirements:** Microfluidic systems require high engineering knowledge and resources.	**Personalized medicine:** Ability to test response to drugs with patient-based 3D models.	**Clinical validity:** The effectiveness of the model in clinical applications is still questionable.	
**Use of dielectrophoresis:** An advanced technology for single-cell measurements.	**Identifying target cells:** The heterogeneous structure of cancer cells can make analysis difficult.	**New treatment approaches:** New treatment methods can be developed by focusing on drug resistance.	**Technological adoption challenges:** Microfluidic devices may not be widely adopted.	[155]
**Drug resistance analysis:** An effective method to detect drug resistance of cancer cells.	**Complex analysis:** Analysis of multidrug-resistant cells can become more complex.	**Clinical tests:** Potential for use in detecting drug resistance at the clinical level.	**Financial challenges:** High-cost instruments and assays can be a barrier to clinical laboratories.	
**Fast results:** High-throughput, single-cell measurements.	**Chip design challenges:** The design and implementation of a dielectrophoresis system can be challenging.	**Personalized treatment:** Individual treatment plans can be developed based on drug resistance.	**Insufficient patient data:** Limited use of real patient samples.	
**Integration of dual technology:** Dielectrophoresis and impedance measurements are used together, achieving more precise results.	**Technological challenges:** Developing devices can be complex and time-consuming.	**Clinical studies:** May enable studies on drug resistance at the clinical level.	**Difficulty of classical treatment approaches:** It may take time to replace existing treatment methods.	[156]
**Drug resistance analysis:** Capacity to detect drug resistance of leukemia cells with high accuracy.	**Non-inclusive patients:** More patient samples may be needed to cover individual differences.	**Treatment compliance:** Treatment changes based on drug resistance can be made more accurate.	**Complex patient samples:** The heterogeneous structure of leukemia cells may affect the accuracy of the analysis.	
**High accuracy:** Counting cells and measuring drug resistance can be achieved with extreme precision.	**High cost of microfluidic devices:** Advanced devices can strain lab budgets.	**New treatment opportunities:** New treatment strategies can be developed to target drug-resistant cells.	**Regulation:** Such new devices may run into regulatory hurdles.	
**Modeling bone marrow angiogenesis:** An opportunity to test the effects of leukemia cells on vessel formation in the bone marrow.	**In vitro limitations:** Complex processes such as angiogenesis can be difficult to fully simulate in vitro.	**Advanced therapeutic research:** Developing new therapeutic approaches by targeting bone marrow angiogenesis.	**Clinical validity:** The extent to which in vitro results can be translated into clinical practice is questionable.	[157]
**Contribution to cancer research:** Ability to examine the interaction of cancer cells with their environment.	**Technological requirements:** Advanced microfluidic devices and analysis techniques are required.	**Drug development:** Drug development studies can be conducted on angiogenesis inhibition.	**Targeting challenges:** Interactions in the microenvironment of cancer cells can be difficult to precisely identify.	
**New therapeutic targets:** Potential for discovery of new targets in cancer therapy.	**Difficulty in clinical transition:** Validation of models in clinical trials may take time.	**Discovery of new biomarkers:** Discovery of new biomarkers involved in the angiogenesis process.	**Cost:** The cost of such advanced testing can be quite high.	

**Table 10:** SWOT analyses of drug testing and imaging BoCs.

Strengths	Weaknesses	Opportunities	Threats	Ref.
**High throughput:** High-throughput drug screening can be performed with single-cell cloning arrays.	**Complexity:** Microfluidic platforms used for high throughput can be complex.	**Personalized medicine:** The potential to develop individual treatment strategies.	**Pressure from traditional testing:** Replacing current standard therapy tests with microfluidic systems may take time.	[194]
**Drug efficacy testing:** An effective system for measuring the effectiveness of cancer treatments.	**Scalability:** Such platforms may be difficult to expand into clinical practice.	**High-throughput drug discovery:** New treatment options for cancer drugs can be discovered.	**Clinical adoption:** The usability of microfluidic platforms in the clinical setting may be uncertain.	
**Individual cell analysis:** Effective analyses can be performed on cancer cells at the single-cell level.	**Technological requirements:** Advanced engineering and investment are required to develop microfluidic devices and systems.	**High throughput analysis:** Big data analysis can be performed on cancer treatments.	**Technological barriers:** Special infrastructure is required for the fabrication and calibration of microfluidic devices.	
**Various treatment protocols:** Allows for testing of different treatment scenarios.	**High cost:** The cost of such advanced systems can be prohibitive for small clinical laboratories.			
**Chemical cytometry:** Microfluidic device that measures peptide degradation, a very sensitive method for analyzing the effect of drug therapy.	**Limited application:** Only studied in AML cells; may be difficult to generalize to broader cancer types.	**Drug research:** Testing AML treatments more efficiently.	**Lack of standards:** Microfluidic technology may not yet have reached clinical standards.	[195]
**Single-cell analysis:** The capacity to perform detailed analyses at the cellular level.	**Technological challenges:** Implementation of microfluidic devices and chemical cytometry can be complex.	**High-precision treatment analysis:** Potential for better treatment decisions in AML treatments.	**Clinical validity:** Microfluidic devices can be difficult to adapt to clinical applications.	
**Precise measurement:** By examining changes in peptide degradation, information about treatment efficacy can be provided.	**Low generalizability:** Its efficacy is questionable across different cell types and treatment scenarios.	**Discovery of new biomarkers:** Biomarkers may emerge to assess the effectiveness of drug therapy.	**The barrier to more traditional methods:** Traditional cell culture and testing methods can be a barrier to microfluidic devices.	
**In-depth information on drug effects:** Information on biochemical changes in cells can be provided.	**Chip design:** Such microfluidic systems are optimized for specific experimental conditions; generalization may be difficult.			
**A 3D microfluidic model:** A 3D modeling of the diffuse large B cell lymphoma microenvironment.	**Limitations of the model:** The fact that the model only works in a laboratory environment may limit its clinical validity.	**New treatment approaches:** More efficient treatment strategies can be developed for rCHOP treatment.	**Biomimetic challenges:** It can be difficult for a 3D environment to reflect all biological dynamics.	[196]
**Treatment effect analysis:** Ability to examine in detail the effects of rCHOP treatment on the microenvironment.	**Limited cell models:** This study examined only a specific treatment type and cell type, limiting generalizability.	**Personalized treatment:** Personalizing the response of different patients to treatment.	**Technological adoption:** Clinical laboratories and hospitals may not easily adopt microfluidic technology.	
**Use of microfluidic platforms:** Using a powerful microfluidic platform for effective treatment testing.	**High cost of devices:** Microfluidic systems can be expensive to install and maintain.	**High-throughput drug screening:** More efficient testing of cancer treatments.	**Clinical validation:** The accuracy of such systems in the clinical setting is questionable.	
**Precise treatment evaluation:** Direct observation of treatment effects can be achieved.	**Time-consuming process:** Setup and analysis of microfluidic systems can be time-consuming.			
**Single-cell-based analysis:** Provides detailed information on treatment sensitivity in multiple myeloma.	**Technological complexity:** Single-cell analyses can be complex and resource-intensive.	**Personalized treatment approaches:** Individual treatment strategies can be developed in the treatment of multiple myeloma.	**Implementation challenges:** Adapting microfluidic systems for clinical use can be complex.	[198]
**Precision therapy assessment:** Provides precise and sensitive information on drug therapy sensitivity.	**Need for a larger patient population:** The model may need to be tested on a larger patient population.	**Discovery of new biomarkers:** Discovery of biomarkers that influence sensitivity to treatment.	**Difficulty of current treatment methods:** It may be difficult to replace traditional treatment methods.	
**High accuracy:** Using single-cell mass accumulation, treatment sensitivity can be determined with high accuracy.	**Time-consuming process:** Single-cell analyses can be time-consuming.	**High-throughput drug testing:** More effective drug screening for multiple myeloma treatments may be possible.	**Clinical approval processes:** It may take time for new technologies to receive clinical approval.	
**Innovative treatment detection method:** Could offer potential new treatment options for multiple myeloma.	**Requirement for large numbers of cell samples:** Analysis of large numbers of cell samples is required.			

## Data Availability

Not applicable.
